# Comparison of short-term clinical outcomes of a diffractive trifocal intraocular lens with phacoemulsification and femtosecond laser assisted cataract surgery

**DOI:** 10.1186/s12886-024-03440-7

**Published:** 2024-04-24

**Authors:** Haokun Qu, Adilamu Abulimiti, Jianheng Liang, Suowang Zhou, Zheming Wu, Yun Chen, Ruihong Ju, Zheng Wang, Rong Xu, Xu Chen

**Affiliations:** 1 Aier Eye Hospital, Jinan University, No. 191, Huanshi Middle Road, Yuexiu District, Guangzhou, Guangdong China; 2 Jinan University, No.601, Huangpu Road West, Guangzhou, China; 3 Department of Ophthalmology, Shanghai Aier Eye Hospital, Shanghai, China; 4 Department of Ophthalmology, Shanghai Aier Qingliang Eye Hospital, Qingpu, Shanghai, China; 5 Department of Ophthalmology & Optometry, SinoUnited Health Clinic, Shanghai, China; 6 Hankou Aier Eye Hospital, Wuhan, China

**Keywords:** Cataract, Intraocular lens, Trifocal, Femtosecond laser, Visual acuity, Refractive outcomes

## Abstract

**Purpose:**

To evaluate short-term visual and refractive outcomes after implantation of a diffractive trifocal intraocular lens (IOL) in cataract patients with phacoemulsification (PHACO) and femtosecond laser assisted cataract surgery (FLACS).

**Setting:**

Department of Ophthalmology, Shanghai Aier Eye Hospital, China.

**Design:**

A retrospective, observational study.

**Methods:**

Patients who underwent cataract surgery combined with Acrysoft IQ PanOptix trifocal IOL implantation were enrolled and divided into three groups: PHACO group, LAstig-FLACS group (astigmatism less then 1D) and HAstig-FLACS group (astigmatism more than 1D). Logarithm of the minimum angle of resolution (logMAR) visual acuity of uncorrected distance (UDVA), intermediate (UIVA), near visual (UNVA), defocus curve, surgically induced astigmatism (SIA) were evaluated in 1 months postoperatively and wavefront aberrations were evaluated in 6 months.

**Results:**

101 eyes of 60 patients were included with 31 eyes in PHACO group, 45 eyes in LAstig-FLACS group and 25 eyes in HAstig-FLACS group. Significant difference was found of internal Strehl Ratio (SR) between PHACO and LAstig-FLACS group (*P* = 0.026). In PHACO group, 79.31%, 86.21%, 72.41% of eyes gain visual acuity LogMAR 0.1 or more in UDVA, UIVA and UNVA, while 83.72%, 93.02%, 93.02% of those in LAstig-FLACS group and 92.00%, 84.00%, 76.00% in HAstig-FLACS group.

**Conclusions:**

Panoptix diffractive trifocal IOL provides satisfied visual outcome in no matter FLACS or PHACO. Besides, trifocal IOL implantation via FLACS can provide a better accumulative visual acuity outcome at all distance than PHACO in 1 month. Femtosecond laser assisted limbal relaxing incisions (FLLRIs) is an excellent way to reduce a patient’s corneal astigmatism.

**Supplementary Information:**

The online version contains supplementary material available at 10.1186/s12886-024-03440-7.

## Introduction

Cataract is a loss of transparency of the lens leading to vision loss and has become the largest cause of blindness worldwide [1]. The most effective treatment for cataract is lens extraction using phacoemulsification (PHACO) devices [1, 2]. At the end of the procedure, an intraocular lens was implanted to restore visual function. Compared to traditional monofocal intraocular lens (IOLs), trifocal IOLs provide good distance, intermediate and near vision at the same time, reducing patients’ dependence on glasses after cataract surgery to meet their reading and living needs [3, 4]. Therefore, trifocal IOLs are currently becoming a choice for presbyopia correction in cataract patients.

The femtosecond laser was first used in refractive surgery and then cataract surgery in 2008 (Femtosecond Laser Assisted Cataract Surgery, FLACS), which was considered a revolutionary innovation in cataract surgery [5]. Advantages of FLACS include shorter phacoemulsification time, less loss of endothelial cells, and more precise capsular and keratotomy incision [6]. More and more clinical studies have focused on evaluating the benefits of FLACS over traditional PHACO cataract surgery with monofocal IOLs [7].

However, whether FLACS combined with trifocal IOLs can achieve a better visual acuity and refractive effect than PHACO combined with trifocal IOLs in the short term has not yet been clearly concluded [8–10]. The aim of our study is to evaluate short-term visual and refractive outcomes after implantation of a diffractive trifocal IOL (AcrySof IQ PanOptix Model TFNT00, Alcon Laboratories, Fort Worth, TX ) in cataract patients with PHACO and FLACS.

## Method

### Study designs and patients

This retrospective observational clinical study was conducted at Shanghai Aier Eye Hospital.

Patients scheduled to undergo cataract surgery from September 2020 to June 2021 were recruited. The study was approved by the Ethics Committee for Human Research at Shanghai Aier Eye Hospital and adhered to the tenets of the Helsinki Declaration. Written informed consent was obtained from each patient. The study included 101 eyes of 60 patients who underwent cataract surgery with the implanted PanOptix trifocal IOL and were enrolled in three groups by different surgical approaches and corneal astigmatism: PHACO, L_Astig_-FLACS (corneal astigmatism less than 1D) and H_Astig_-FLACS groups (corneal astigmatism more than 1D). Inclusion criteria are as follows: (1) lens opacity; (2) regular corneal astigmatism ≤ 3D; and (3) Alpha angle < 0.5 mm and Kappa angle < 0.3 mm. Exclusion criteria are as follows: (1) severe corneal clouding that prevents passage of the laser; (2) severe systemic disease (e.g. diabetes); (3) other eye diseases such as glaucoma, age-related macular degeneration, etc.; (4) prior history of refractive surgery. Due to only 2 eyes with corneal astigmatism more than 1D, the PhACO group was not divided to subgroups with low or high astigmatism which was discussed detailly in the below section.

### Pre- and post-operative evaluation procession and data collection

Preoperative evaluation was performed on all patients including complete anterior segment examination with slit-lamp biomicroscopy, applanation tonometry, fundus examination, corneal specular microscopy and topography as well as iTrace ray tracing aberrometer (Tracey Technologies, Houston, USA). IOLMaster 700 (Carl Zeiss Meditec, Jena, Germany) was also used to measure axial length and calculate IOL power using Barrett Universal II formula with a refractive target for emmetropia. For all patients with corneal astigmatism more than 1D, we recommended femtosecond laser assisted limbal relaxing incision (FLLRI) to correct the astigmatism, and most of the patients agreed to this plan, with only a small number of patients declining it due to cost considerations. All intraocular lenses were implanted in the capsular bag and avoid postoperative complications appeared.

The main outcome measures were Logarithm of the minimum angle of resolution (logMAR) visual acuity of uncorrected distance (UDVA), intermediate (UIVA), near visual (UNVA), defocus curve, surgically induced astigmatism (SIA). All patients were assessed for logMAR visual acuity (UDVA, UIVA, UNVA), defocus curve and SIA in 1month and logMAR visual acuity (UDVA, UIVA, UNVA), wavefront aberrations in 6 months postoperatively.

### FLACS and PHACO procedure

Every patient accepted the standard surgical procedure. One experienced surgeon (C.X.) performed all surgical procedures with the same equipment. In order to avoid potential effects due to differences in operating habits [11, 12], all procedures are performed with the operator’s dominant (right) hand. Before surgery, topical levofloxacin 0.5% was given to all patients 4 times daily for 1 day. Pupil dilation was achieved with the instillation of 1 drop of tropicamide every 15 min, 3 times before surgery. In FLACS group, the clear corneal incision and a sideport, capsulotomy and lens fragmentation were made using the LenSx platform (Alcon Laboratories, Inc., Fort Worth, TX, USA). The 2.4 mm main corneal incision at 135 degree site and a 1.0 mm side port at 45 degree site were made manually and sleeve using the Alcon Centurion system (Alcon Laboratories, Inc., Fort Worth, TX, USA). For patients with preoperative corneal astigmatism of more than 1.0 D, manual LRI in PHACO group and FLLRIs in FLACS group will be performanced and planned with LRI Online Calculator (https://www.lricalculator.com/). FLLRIs possess a limbal center and a diameter of 8.5 mm, with paired symmetrical arcs. The FL platform integral optical coherence tomography (OCT) was used to measure the corneal pachymetry, and the arcs were configured to be intrastromal, non-penetrating, and to have a depth of 20–80%.

### PanOptix intraocular lens

PanOptix’s Trifocal IOL is an aspheric hydrophobic intraocular lens with a blue filter and 6.0 mm optical zone, consisting of a large 4.5 mm diffractive zone and 15 diffractive zones and an outer refractive edge. There are three focal points from far to intermediate and near distances, splitting incident light to produce mid- and near-range diopters of 2.17 diopters (D) and 3.25 D, respectively. Therefore, it offers the best close reading distances of 60 cm and 42 cm. This novel diffractive structure provides high light utilization, delivering 88% of the light that simulates a 3.0 mm pupil size to the retina. This light energy is split 25% for nearsightedness and intermediate vision, and 50% for farsightedness.

### Statistical analysis

Statistical analysis was performed by SPSS 26.0 for Windows (SPSS Inc., Chicago, Illinois, USA). We used descriptive statistics to assess the value of the data. To determine the normality of data distribution, Kolmogorov-Smirnov test was carried out. Kruskal-Wallis and one-way ANOVA test was chosen to assess between-group differences between the FLACS and PHACO groups in normal distribution variables, Wilcoxon and Mann-Whitney U test for non-normal distribution ones and qualitative information. For normally distributed data, continuous and categorical variables were described as mean ± standard deviation (SD) and number and percentage (%) and *P* < 0.05 was considered as statistically significant. A total of 2 eyes received manual LRI and 25 eyes received FLLRI in our study.

## Result

### Baseline characteristics

Table 1 shows the baseline characteristics. 31 eyes from 21 patients were included in the PHACO group, with a mean age of 57.94 ± 12.31 years. 45 eyes from 31 patients were included in the L_Astig_-FLACS group, with a mean age of (54.37 ± 10.74) years. 25 eyes from 21 patients were included in the H_Astig_-FLACS group, with a mean age of 55.48 ± 11.93 years. All patients completed the follow-up during the 1 month. No eye was excluded from the analysis because of the postoperative complications. Significant differences were not found in age, axial length (AL), anterior chamber depth (ACD), flat K and steep K but found in astigmatism between 3 groups.

**Table 1:** Baseline characteristics

Characteristics	PHACO	L_Astig_-FLACS	H_Astig_-FLACS	P-Value
Eye.no(%)	31(30.7)	45(44.5)	25(24.8)	/
Patients.no(%)	21(33.9)	31(50)	21(33.9)	/
Age(years)	57.94 ± 12.31	58.37 ± 10.74	55.48 ± 11.93	0.434
AL(mm)	26.29 ± 2.65	26.31 ± 2.20	26.35 ± 2.40	0.898
ACD(mm)	3.44 ± 0.33	3.25 ± 0.38	3.31 ± 0.35	0.086
Astignatism(D)*	0.75 ± 0.53*	0.54 ± 0.25*	1.40 ± 0.41*	0.000*
Flat K(D)	42.34 ± 2.84	42.21 ± 2.21	41.89 ± 2.48	0.578
Steep K(D)	43.10 ± 3.00	42.74 ± 2.46	43.29 ± 2.42	0.357

### Visual acuity

**Fig. 1:**
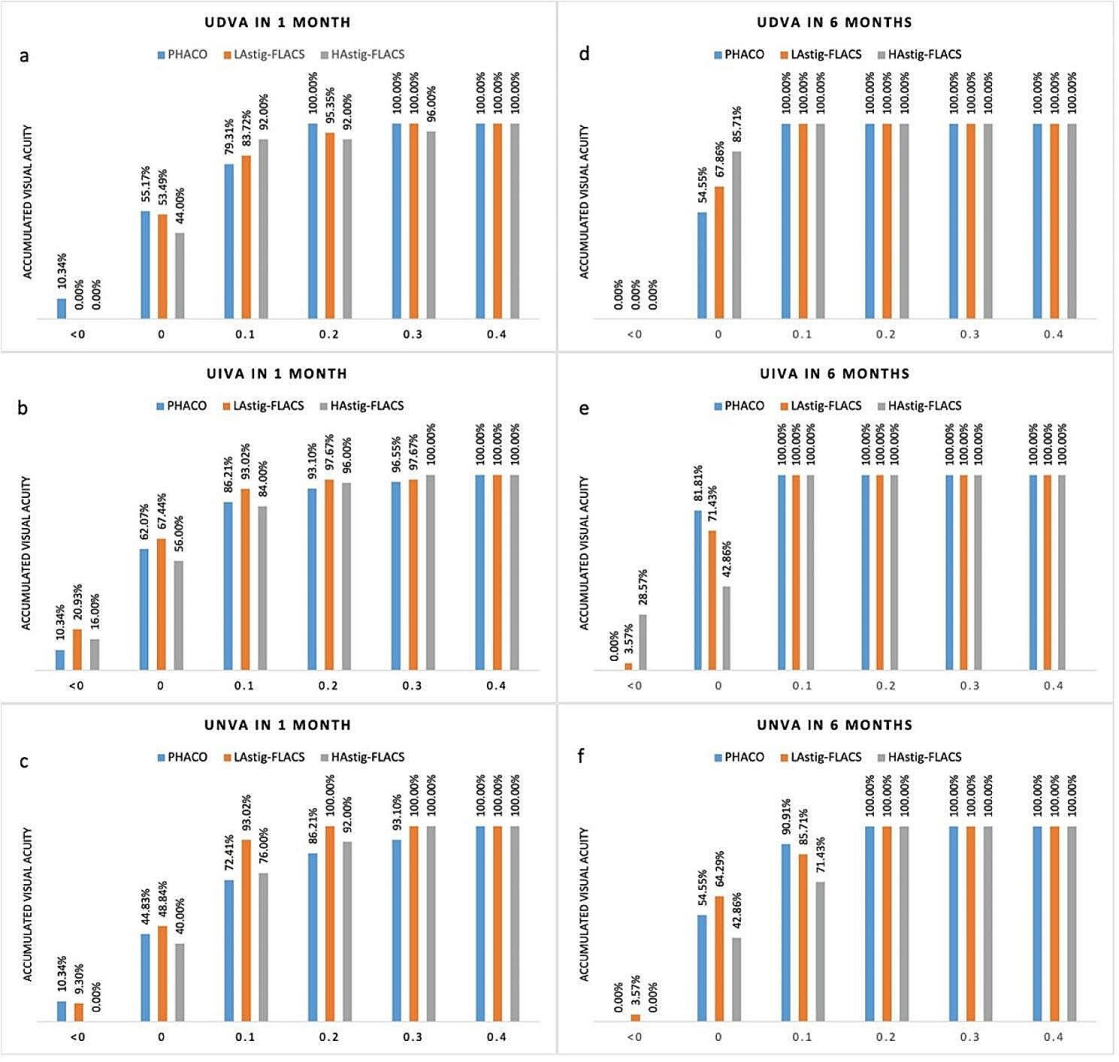
Comparison of accumulative visual acuity (logMAR) between UDVA (**a**, **d**), UIVA (**b**, **e**) and UNVA (**c**, **f**) group at 1 month and 6 months

Figure 1 shows the comparison of accumulative visual acuity at 1 month and 6 months between 3 groups. In PHACO group at 1 month, 79.31%, 86.21%, 72.41% of eyes achieved visual acuity LogMAR 0.1 or more in UDVA, UIVA and UNVA, while 83.72%, 93.02%, 93.02% of those in L_Astig_-FLACS group and 92.00%, 84.00%, 76.00% in H_Astig_-FLACS group. At 6 months, 100% of eyes in all groups achieved LogMAR 0.1 or more in UDVA, UIVA. No significant difference were found between 3 groups in UDVA, UIVA, UNVA in both 1month and 6 months (*P* > 0.05) (Tables 2 and 3).

**Table 2:** Uncorrected visual acuity (logMAR) at 1 month postoperatively

	PHACO	L-FLACS	H-FLACS	P Value
UDVA	0.06 ± 0.09	0.07 ± 0.09	0.08 ± 0.12	0.843
UIVA	0.05 ± 0.11	0.03 ± 0.09	0.05 ± 0.10	0.454
UNVA	0.09 ± 0.13	0.05 ± 0.07	0.09 ± 0.10	0.304

**Table 3:** Uncorrected visual acuity (logMAR) at 6 months postoperatively

	PHACO	L-FLACS	H-FLACS	P-Value
UDVA	0.04 ± 0.05	0.03 ± 0.04	0.01 ± 0.04	0.410
UIVA	0.02 ± 0.04	0.02 ± 0.05	0.03 ± 0.08	0.719
UNVA	0.05 ± 0.07	0.05 ± 0.08	0.08 ± 0.09	0.675

### Defocus curves

**Fig. 2:**
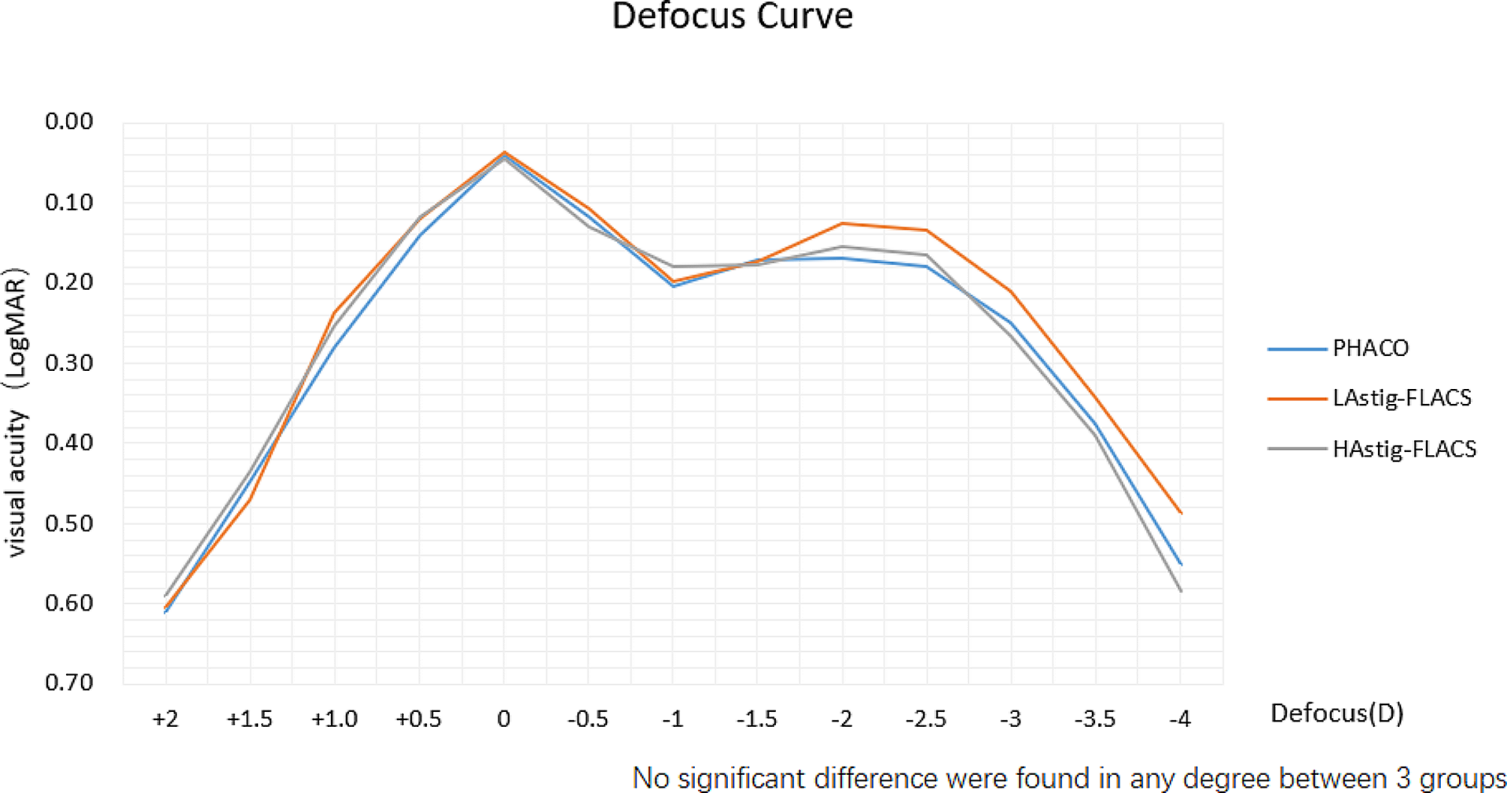
Defocus curve at 1 month postoperatively

Figure 2 shows the mean defocus curves at 1 month postoperatively. The best visual acuity results were observed in all groups at 0.00 D defocus equivalent to distance vision. A second peak was observed at -1.5D in PHACO group while − 2.0D in L_Astig_-FLACS and H_Astig_-FLACS group, corresponding to good median vision. The third peak were observed in both group at -2.5 D defocus corresponding to good near vision. All groups showed the similar defocus curve.

### Spherical equivalence and residual astigmatism at 1 month

**Fig. 3:**
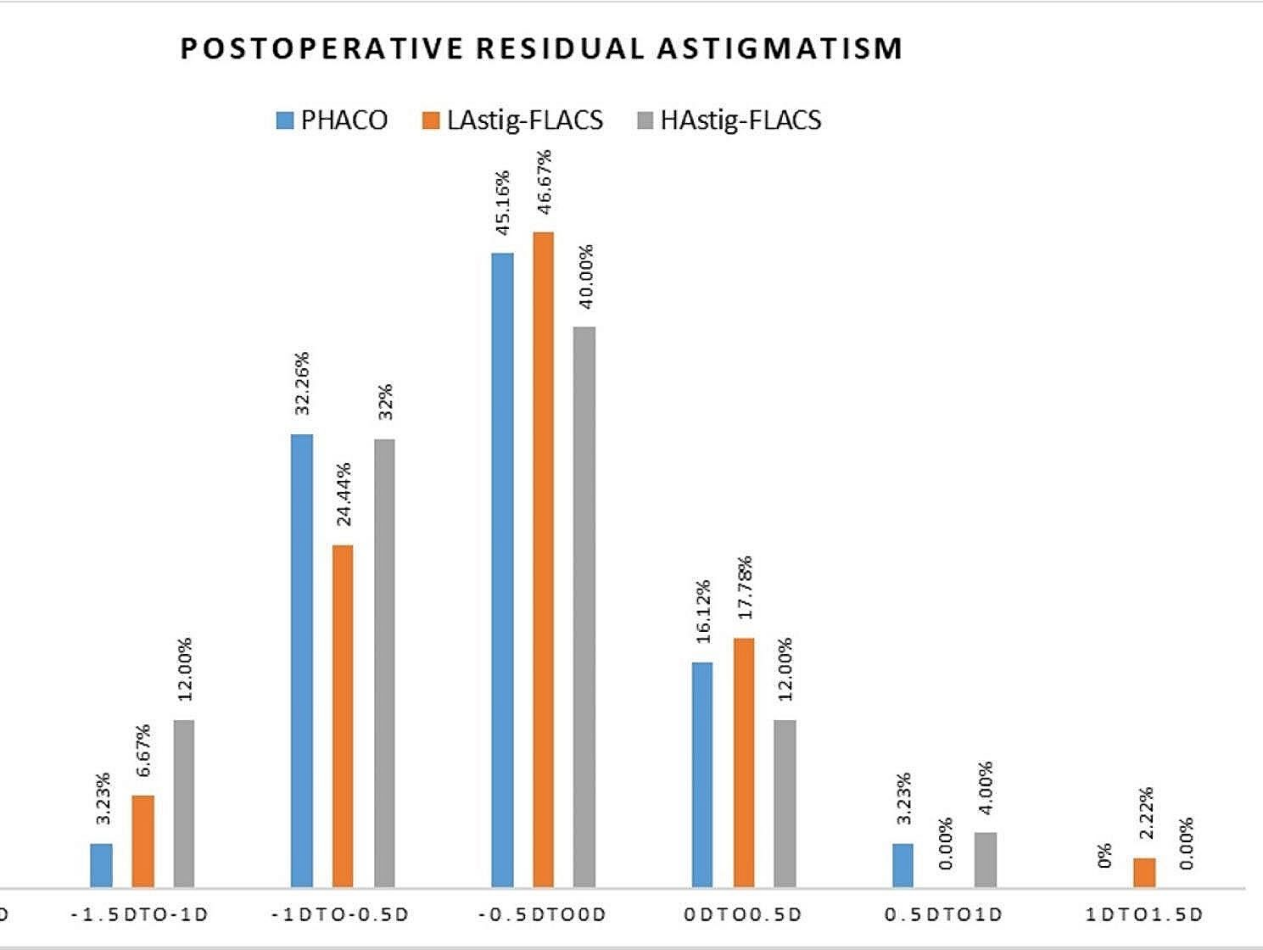
Comparison of postoperative residual astigmatism between 3 groups at 1 month

There was no statistically significant reduction in Spherical Equivalence (SE) after surgery (*P* > 0.05). Table 4 shows the comparison of preoperative and postoperative SE and postoperative residual astigmatism. Figure 3 shows the postoperative residual astigmatism between 3 groups at 1 month. The percentage of postoperative residual astigmatism within ± 0.5D were 61.28%, 64.44% and 52% in PHACO, L_Astig_-FLACS and H_Astig_-FLACS group, and within ± 1.0D were 96.77%, 88.89% and 88% in each group. There was no significant difference of postoperative SE and residual astigmatism between two groups (*P*>0.05).

**Table 4:** Postoperative Spherical Equivalence (SE) and postoperative residual astigmatism in 1 month

	PHACO	L_Astig_-FLACS	H_Astig_-FLACS	P
Postoperative SE(D)	-0.19 ± 0.40	-0.28 ± 0.48	-0.27 ± 0.71	0.774
Postoperative RA(D)	-0.45 ± 0.41	-0.48 ± 0.50	-0.53 ± 0.51	0.811

### Wavefront aberrations at 6 months

**Fig. 4:**
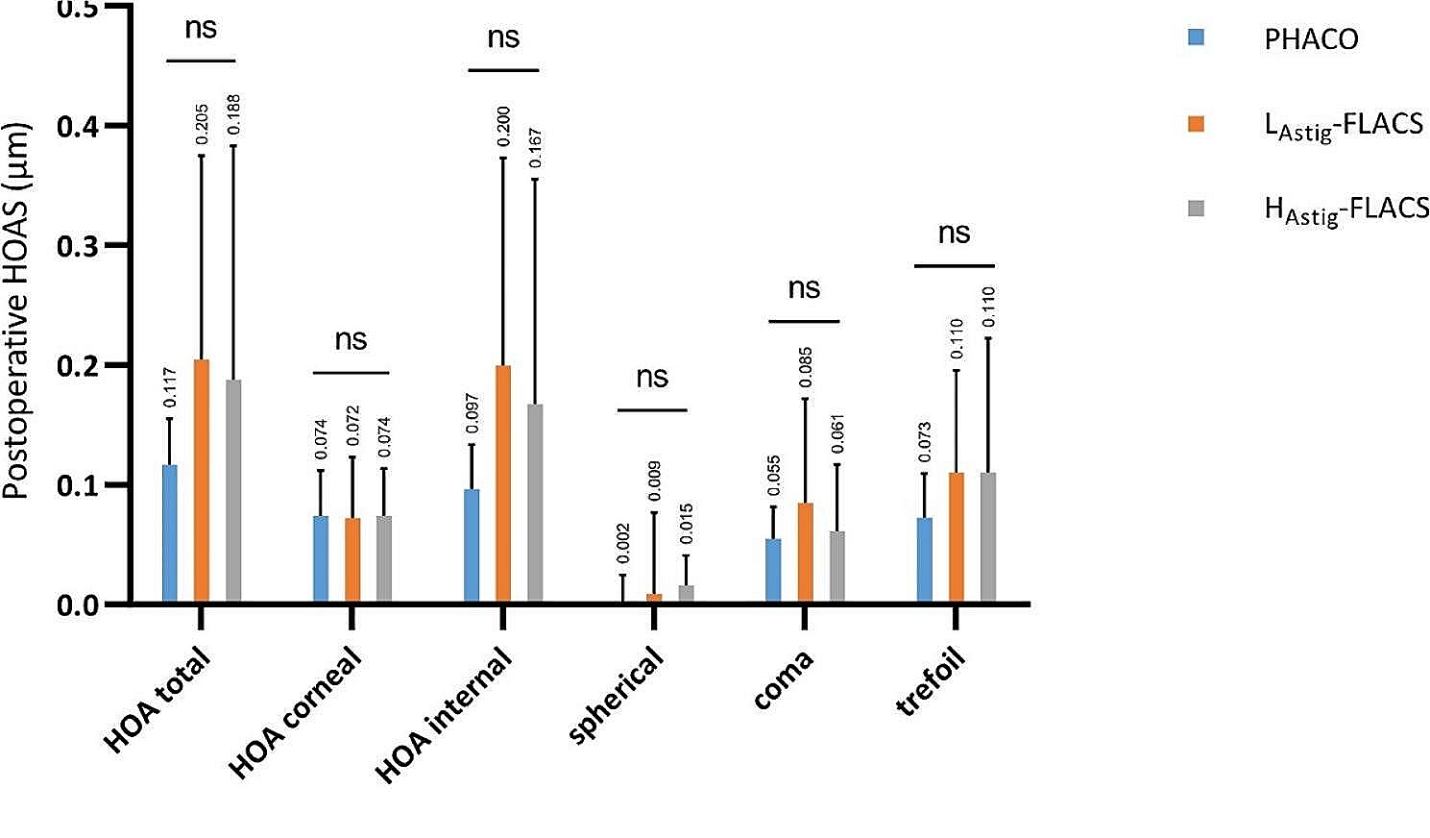
Distribution of HOAs, coma, spherical aberration and trefoil with the pupil diameter of 4 mm in 3 groups at 6 months

**Fig. 5:**
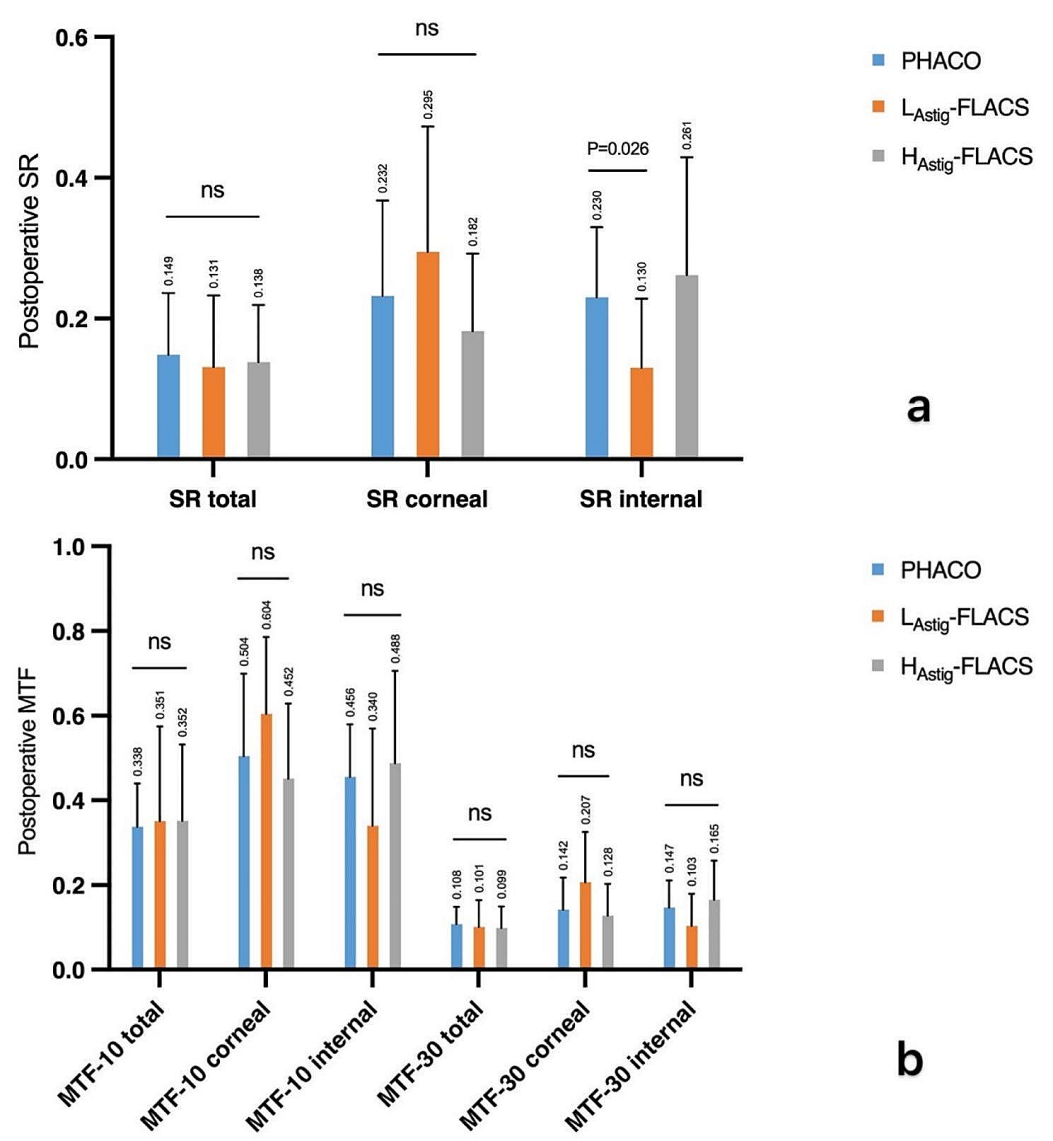
Comparisons of SR (**a**) and MTF (**b**) in 10 cpd and 30 cpd with the pupil diameter of 4 mm between 3 groups

Figure 4 shows the distribution of wavefront aberrations, including total eye aberrations, corneal aberrations, internal aberrations and higher order aberrations (coma, trefoil, and spherical aberrations) with the pupil diameter of 4 mm in 3 groups at 6 months. The incidence of postoperative ocular aberrations, including total higher-order aberrations (HOAs) (*P* = 0.31), corneal HOAs (*P* = 0.74), internal HOAS (*P* = 0.12), coma (*P* = 0.71), trefoil (*P* = 0.40), and spherical aberration (*P* = 0.45), did not significantly different (*P*>0.05) between the 3 groups. Figure 5 shows the comparisons of SR (a) and MTF (b) in 10 cpd and 30 cpd with the pupil diameter of 4 mm between 3 groups. The MTF value describes the relationship between the contrast of the image and the quality of the optical system at different spatial frequencies. A higher MTF value means a clearer image and better visual quality. No significant difference (*P*>0.05) were found in MTF value between the two groups in both 10 cpd and 30 cpd. Significant difference (*P* = 0.026) were found in internal SR between PHACO and L_Astig_-FLACS groups.

### Surgically induced astigmatism (SIA)

**Fig. 6:**
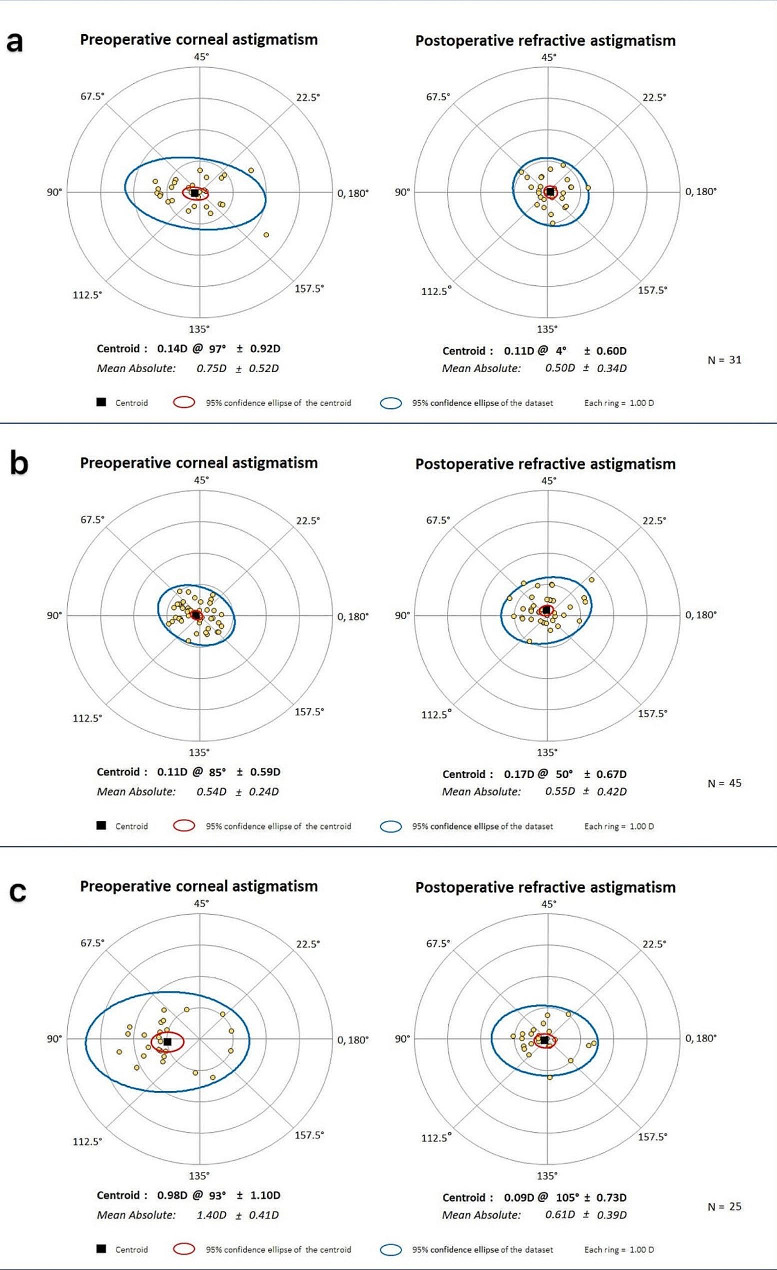
Preoperative corneal astigmatism and postoperative refractive astigmatism between PHACO (**a**), L_Astig_-FLACS (**b**) and H_Astig_-FLACS group (**c**) at 1 month

The surgically induced astigmatism (SIA) is defined as the amount and axis of the astigmatism that was induced by the surgery [13]. Total astigmatism values preoperatively and 6 months postoperatively were analysed using the Alpins method [14], where preoperative and postoperative K values and their axes were used to assess the effective change in astigmatism values, taking into account the change in the axis of astigmatism. Regarding to refractive predictability, the achievement of the target apparent refraction is measured by calculating the absolute difference between the target refraction and the postoperative equivalent spherical lens. Figure 6 shows the Preoperative corneal astigmatism and Postoperative refractive astigmatism between PHACO (a), L_Astig_-FLACS (b) and H_Astig_-FLACS group (c). In the PHACO group, the mean values of preoperative corneal astigmatism and postoperative refractive astigmatism were 0.75 ± 0.52D and 0.50 ± 0.34D. And those were 0.54D ± 0.24D and 0.55D ± 0.42D in L_Astig_-FLACS group, while 1.40D ± 0.41D and 0.61D ± 0.39D in H_Astig_-FLACS group. No statistical difference between the three groups of postoperative refractive astigmatism (*P* = 0.578). Postoperative residual astigmatism was significantly reduced in the H_Astig_-FLACS group compared to preoperative astigmatism (*P* < 0.0001).

### Postoperative complications

No postoperative complications such as posterior capsular opacification, macular edema were noted in all patients during the follow up period.

## Discussion

Improving visual acuity is the main goal of cataract surgery. The femtosecond laser automated the four initial manual steps performed in cataract surgery [15] to reduce the amount of energy required for PHACO. Although there are theoretical benefits to surrounding structures from reduced ultrasonic emulsion energy, there is still debate about the effectiveness of FLACS in this regard [16].

The goal of this study was to analyze and evaluate short-term visual and refractive outcomes after implantation of a diffractive trifocal IOL in cataract patients with PHACO and FLACS. Existing literature has also focused on comparing whether there is a significant difference in postoperative visual acuity outcomes between the two procedures. Berk et al. reported no statistical significance in visual outcomes between the two groups at 3 weeks post-cataract surgery in an analysis of 1838 eyes [17]. In our study, mean postoperative logMAR UDVA, UIVA, UDVA in PHACO, L_Astig_-FLACS and H_Astig_-FLACS group all achieved better than 0.1 no matter at 1 month or 6 months, which means that Panoptix trifocal IOL also provides patients with good medium distance vision and near vision after surgery in a short term. Our results are consistent with those reported by most authors evaluating the same trifocal IOLs [18–20]. When our results are compared with those obtained with other IOLs, such as monofocal IOLs or bifocal IOLs, the advantages of the diffraction IOLs evaluated are obvious [21–23]. It can be easily explained by higher order aberrations caused by refractive multifocal IOLs compared to diffraction models [21, 23]. Specifically, it is known that rotating asymmetrical refractive multifocal IOLs can cause a lot of primary coma, which may limit the visual acuity of the implant [21, 23].

On the other hand, recent studies have focused on the value of FLACS in HOA control. FLACS has been reported to result in lower HOAs than PHACO [24]. Jin Ah Lee et al. found that astigmatic change was more predictable in the femtosecond laser–assisted cataract surgery group. Internal aberrations, including total RMS, tilt, and RMS HOAs, were lower in the femtosecond group [25]. In our study, no significant differences were found total coma, total trefoil, and total spherical aberration, which is inconsistent with recent research findings. Zhong et al. reported that FLACS demonstrated a significantly lower root mean square of total internal aberration (*P* = 0.004), HOAs (*P* = 0.034), tilt (*P* = 0.049), coma (*P* = 0.004), and spherical aberration (*P* = 0.014) [26]. A lower value of IOL tilt, decentration, and internal aberrations means a higher visual qualification. We consider that the main cause of this error is the difference in pupil diameter. In our study, subjects were examined with a pupil diameter of 4 mm, whereas other studies were examined with 5 mm or more. Pupil size also affects total HOAs, MTF values and SR [27]. According to previous studies, HOAs become larger with larger pupil sizes, leading to changes in SR and MTF values [28, 29].

Our study showed the significant difference of internal SR between PHACO and L_Astig_-FLACS group (*P* < 0.05). Other MTF and SR in each group did not show the significant difference, which is consistent with existing research findings [16, 25, 26]. SR and MTF is an useful parameter of optical quality, which is closely associated with aberrations [30]. With a 4 mm pupil, corneal aberration has little effect on the results, which may be closer to the true optical quality of the IOL [31]. Liu et al. [32] found that SR decreased with increasing AL, especially when AL ≥ 28 mm. This indicates that there is more intraocular light scattering when the AL increasing. But our study did not show the difference between PHACO and L_Astig_-FLACS group. Some previous studies showed higher SR and MTF values at all frequencies in the femtosecond group [32, 33], which are partly similar to our study. Although internal SR appeared to be statistically different in our study, considering that no significant difference in visual acuity was seen between all the groups at 6 months of age, the difference may not be considered clinically valuable. The truth is that the reasons for the discrepancy continue to baffle us. Some studies speculate that the SIA of PHACO and FLACS are different thus leading to differences in MTF and SR [34]. To date, studies investigating HOAs and objective visual quality have produced ambiguous results and more clinical investigations are needed [26].

The near visual outcomes obtained in our study were excellent, with 82.47% of eyes achieving a logMAR UNVA of 0.1 or better, 93.81% of eyes achieving 0.2 or better, and 97.93% eyes achieving 0.3 or better. The mean postoperative logMAR UNVA in all the patients was 0.07 when measured at 40 cm. This result is equivalent to that reported by Mojzis et al. [35]. The intermediate visual effects obtained in our study are also very pleasing, with 88.65% of eyes achieving a logMAR UIVA of 0.1 or better, 97.94% of eyes achieving 0.3 or better, and all eyes achieving 0.4 or better. The mean postoperative logMAR UIVA was 0.04 when measured at 66 cm. Similar UIVA outcomes have been reported by other authors for the same trifocal IOL [18, 36, 37]. Compared to the results of Ma et al’s [38] study of trifocal IOL implantation via FLACS, they applied more satisfactory and stable UNVA, UIVA and UDVA results. The source of the difference may be related to the follow-up time. As in this study we only observed post-operative visual acuity at 1 month postoperatively, while their team observed visual acuity at 1, 3 and 6 months postoperatively [38]. Several other factors may contribute to this difference, such as differences in patient samples (e.g. age, AL) or examination protocol. In the meantime, it can be seen in our results that in PHACO group 79.31%, 86.21%, 72.41% of eyes gain visual acuity LogMAR 0.1 or more in UDVA, UIVA and UNVA, while 83.72%, 93.02%, 93.02% of those in L_Astig_-FLACS group and 92.00%, 84.00%, 76.00% in H_Astig_-FLACS group, which mean FLACS group achieved better accumulative visual acuity outcome at all distance than PHACO group.

The defocus curve is an important indicator for evaluating the performance of multifocal IOLs, mainly reflecting the patient’s continuous visual range [38]. The 1-month postoperative defocus curve of this study showed two peaks at 0D and − 2.5D in FLACS group while showed three peaks at 0D, -1.5D and − 2.5D in PHACO group, but the transition of these curves was smooth in the middle. The peaks indicates that the patient’s distance and near vision is sufficiently clear after surgery, and a flat change indicates a stable and clear transition between distance and near. This is mainly because the PanOptix trifocal IOL increased + 2.17D vision and increased + 3.25D near vision, which is consistent with the finding of Poyales et al. [39].

Femtosecond laser-assisted corneal surgery offers a more precise and safer approach to eye surgery, as the femtosecond laser provides a more precise incision position and improve the prediction of corneal shape [40] which may reduce SIA. With advances in cataract surgery techniques, there are more ways to reduce corneal astigmatism such as clear corneal incision (CCI) at the steepest meridian, opposed clear corneal incision (OCCI), manual LRI, FLLRIs and toric IOL implantation [41]. It has been suggested that CCI, paired OCCI, and Toric IOLs implantation should be prioritized for patients with low, intermediate, and high astigmatism [42]. At the time of our study, since the toric version of the Panoptix lens was not yet available in China, manual LRI and FLLRIs were chosen to address astigmatism in the patients. In our study, the effect of astigmatism on the PHACO group will not be discussed for the following reasons. Firstly, the volume of eyes with corneal astigmatism more than 1D was very low. (Only 2 eyes in the H_Astig_-PHACO group), which led to possible bias in the statistics. Even if we perform statistical analysis with the PHACO group by using two groups (L_Astig_-PHACO and H_Astig_-PHACO), there is no difference between the results and the current results. (see Supplementary Material for details).

Our study showed the mean values of preoperative corneal astigmatism and postoperative refractive astigmatism were 0.75 ± 0.52D and 0.50 ± 0.34D in PHACO group. And those were 0.54D ± 0.24D and 0.55D ± 0.42D in L_Astig_-FLACS group, while 1.40D ± 0.41D and 0.61D ± 0.39D in H_Astig_-FLACS group. Postoperative refractive astigmatism prediction error in two FLACS group were both smaller than that in PHACO group, which means the accuracy for femtosecond laser-constructed corneal wounds may better than the manual keratome. However, in the absence of a meaningful statistical analysis, our conclusion cannot yet be proven. Among the available studies, a mean SIA was 0.35 ± 0.67 D for the femtosecond laser-constructed corneal wounds and 0.901 ± 0.882 D for the manual keratome (*p* = 0.015) were found in the study of Shaheen MS et al. [13], which may support our conclusions. But In another series of 48 eyes cataract surgery (20 FLACS and 28 manual cataract surgery) [14], the mean SIA in laser and the manual group at 3 months was 0.60 ± 0.73D and 0.37 ± 0.92 D respectively (*p* = 0.318). Errors may be due to operator proficiency, differences in measurement equipment and conditions and especially differences in follow-up time. Besides, two studies (including 100 eyes) [43] compared surgically induced astigmatism (SIA) after FLACS and PHACO and these data showed no significant difference between the two groups. Patients in our study with high astigmatism were also treated with manual LRI and FLLRI, which provide safe and moderately effective corneal astigmatism correction in cataract surgery [44, 45]. These findings support that there was no difference in absolute postoperative astigmatism error between the two groups.

LRI is one of the safest surgical techniques in cataract surgery for the treatment of corneal astigmatism, with a low probability of intraoperative and postoperative complications [44, 46, 47]. In addition, LRI is also safely used for multifocal IOLs with minimal impact on the final postoperative spherical equivalent [48], eliminating the need to change the IOL calculation method. The result is similar to ours. Several studies have shown that FLLRIs provides a higher correction index and a smaller variance vector than manual LRI [44, 49]. Therefore, we believe that FLLRIs is an excellent way to reduce astigmatism in patients, which is similar to the conclusions of the existing literature [50].

We evaluated the astigmatism issue in FLACS and PHACO combined with trifocal IOL implantation in our study, especially focused on manual LRI and FLLRIs effects. Only one trifocal IOL was enrolled in our study that excludes data bias due to the different design of trifocal IOLs, allowing a better comparison of the impact of FLACS with or without LRI and PHACO on visual results. This study also included an analysis of MTF and SR, two visual quality metrics that have been relatively little reported in trifocal IOLs. We confirmed that the accumulative UNVA, UIVA and UDVA of FLACS combined with trifocal IOL implantation was superior to PHACO combined with trifocal IOL implantation. What’s more, Panoptix diffractive trifocal IOL provides good uncorrected distance, intermediate, and near acuity in both PHACO and FLACS group were also be confirmed. This data can be used to better guide the surgeon in selecting the most appropriate surgical option when implanting trifocal IOLs in cataract patients.

However, there are still several limitations to this study. First, it is a small single-center study and there may be bias in the collection of information. Secondly, the subjects included in this study were predominantly middle-aged and elderly patients. It would be beneficial to enhance the study in younger patients to further validate the effectiveness of FLACS combined with trifocal IOL implantation in younger patients. Thirdly, Alpin astigmatism vector calculations were not used to analyze differences in SIA. It is less accurate to compare the effect of astigmatism correction simply by the size of astigmatism. Finally, patients were followed up for only 6 months in this study and all data were taken from one time point, with no data comparison between time periods. The longer the follow-up period, the more stable and reliable the data will be.

## Conclusion

Panoptix diffractive trifocal IOL provides satisfied visual outcome, including good uncorrected distance, intermediate, and near acuity in no matter FLACS or PHACO combined with FLLRIs and trifocal IOL implantation. Besides, trifocal IOL implantation via FLACS can provide a better accumulative visual acuity outcome at all distance than PHACO in 1 month. FLLRIs has no significant effect on postoperative spherical equivalence and it is an excellent way to reduce a patient’s corneal astigmatism.

### Electronic supplementary material

Below is the link to the electronic supplementary material.

Supplementary Material 1

## Data Availability

The datasets generated and/or analysed during the current study are not publicly available but are available from the corresponding author, X Chen, upon reasonable request.
