# Supplementary material for: Comparison of short-term clinical outcomes of a diffractive trifocal intraocular lens with phacoemulsification and femtosecond laser assisted cataract surgery
Source: BMC Ophthalmol. 2024 Apr 24;24:189. doi: 10.1186/s12886-024-03440-7 (PMC11040763; doi:10.1186/s12886-024-03440-7)

Supplemental Table 1 Baseline characteristics

| characteristics | LAstig-PHACO | HAstig-PHACO | L_Astig_-FLACS | H_Astig_-FLACS | P-Value |
| --- | --- | --- | --- | --- | --- |
| Eye.no(%) | 23(22.8) | 8（7.9） | 45(44.5) | 25(24.8) | / |
| Age(years) | 60.87±8.42 | 49.5±17.81 | 58.37±10.74 | 55.48±11.93 | 0.165 |
| AL(mm) | 26.33±2.79 | 26.17±2.39 | 26.31±2.20 | 26.35±2.40 | 0.971 |
| ACD(mm) | 3.42±0.31 | 3.52±0.4 | 3.25±0.38 | 3.31±0.35 | 0.144 |
| Astignatism(D)* | 0.52±0.31* | 1.43±0.46* | 0.54±0.25* | 1.40±0.41* | 0.000* |
| Flat K(D) | 42.26±3.04 | 42.57±2.34 | 42.21±2.21 | 41.89±2.48 | 0.775 |
| Steep K(D) | 42.78±3.16 | 43.99±2.41 | 42.74±2.46 | 43.29±2.42 | 0.401 |
|  | | | | | *P<0.05 |

Supplemental Table 2 Uncorrected visual acuity (logMAR) and residual astigmatism in 1 month postoperatively

|  | LAstig-PHACO | HAstig-PHACO | L-FLACS | H-FLACS | P Value |
| --- | --- | --- | --- | --- | --- |
| UDVA | 0.07±0.09 | 0.02±0.11 | 0.07±0.09 | 0.08±0.12 | 0.391 |
| UIVA | 0.05±0.10 | 0.06±0.14 | 0.03±0.09 | 0.05±0.10 | 0.753 |
| UNVA | 0.11±0.13 | 0.05±0.15 | 0.05±0.07 | 0.09±0.10 | 0.060 |
| Postoperative RA(D) | -0.45±0.45 | -0.47±0.28 | -0.48±0.50 | -0.53±0.51 | 0.196 |

*At 6 months postoperatively, only 2 eyes in the H_Astig_-PHACO group, which unable to perform statistical analysis.

Supplemental Figure 1 Defocus curve at 1 month postoperatively


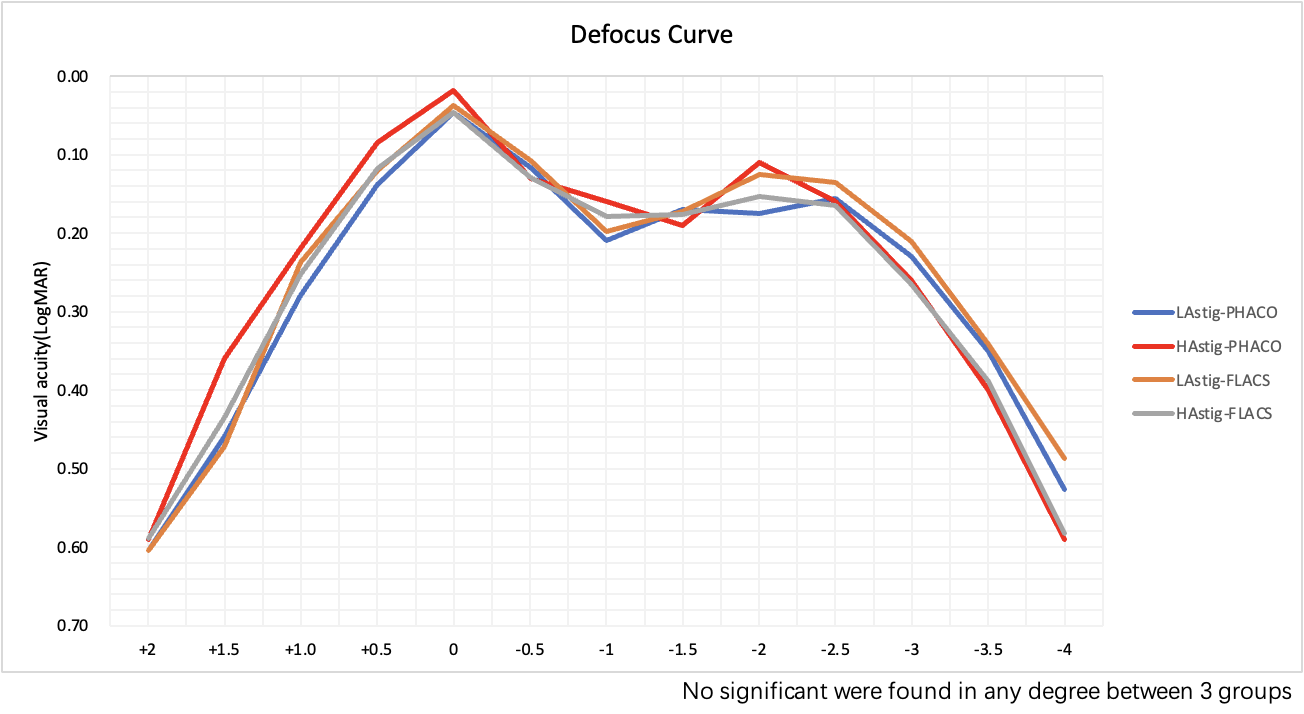


Supplemental Figure 2 Preoperative corneal astigmatism and Postoperative refractive astigmatism between L_Astig_ -PHACO (a), H_Astig_-PHACO (b), L_Astig_-FLACS (c) and H_Astig_-FLACS group (d) at 1 month


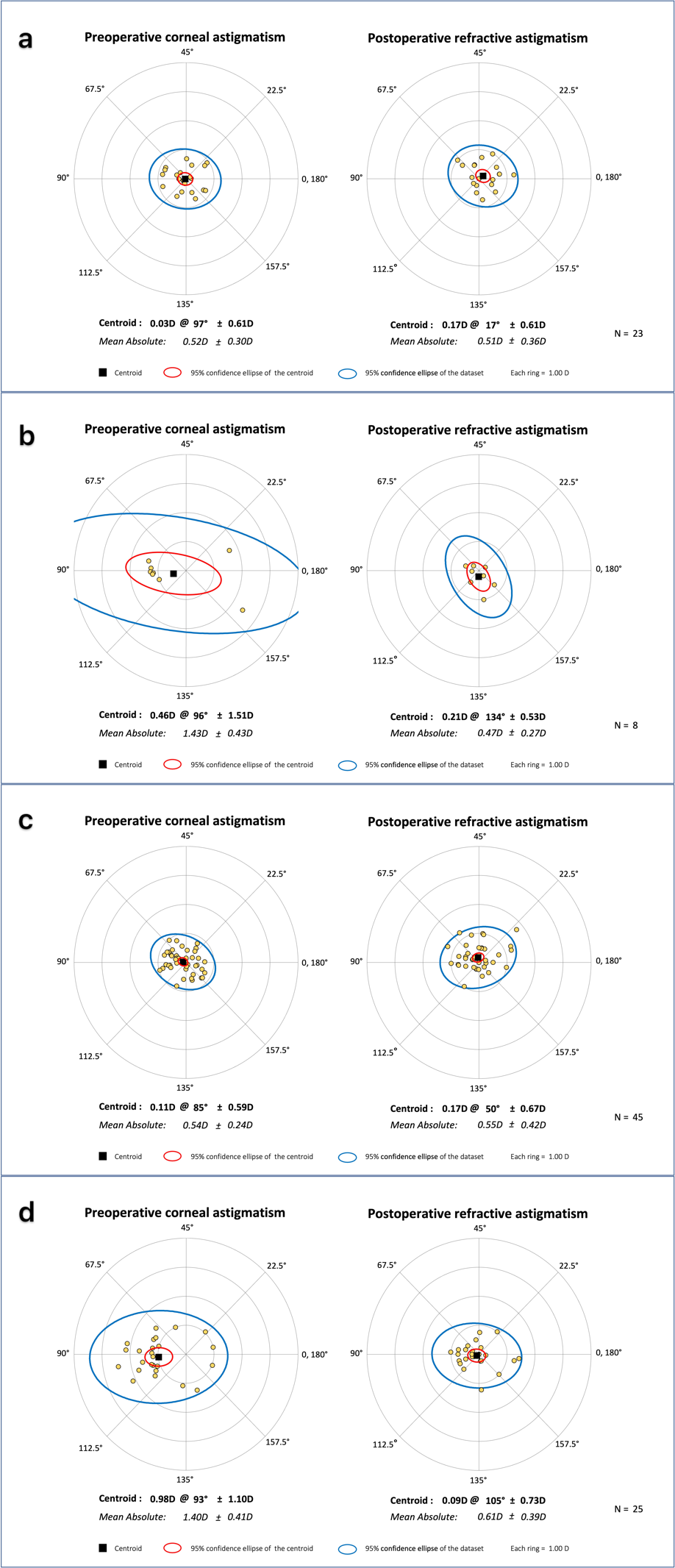

Supplement: Supplementary file 1 — Supplementary Material 1 [file 12886_2024_3440_MOESM1_ESM.docx]
